# EnhFFL: A database of enhancer mediated feed-forward loops for human and mouse

**DOI:** 10.1093/pcmedi/pbab006

**Published:** 2021-04-14

**Authors:** Ran Kang, Zhengtang Tan, Mei Lang, Linqi Jin, Yin Zhang, Yiming Zhang, Tailin Guo, Zhiyun Guo

**Affiliations:** School of Life Sciences and Engineering, Southwest Jiaotong University, Chengdu 610031, China; School of Life Sciences and Engineering, Southwest Jiaotong University, Chengdu 610031, China; School of Life Sciences and Engineering, Southwest Jiaotong University, Chengdu 610031, China; School of Life Sciences and Engineering, Southwest Jiaotong University, Chengdu 610031, China; School of Life Sciences and Engineering, Southwest Jiaotong University, Chengdu 610031, China; School of Life Sciences and Engineering, Southwest Jiaotong University, Chengdu 610031, China; College of Medicine, Southwest Jiaotong University, Chengdu 610031, China; School of Life Sciences and Engineering, Southwest Jiaotong University, Chengdu 610031, China

**Keywords:** database, enhancer, miRNA, transcription factor, feed-forward loop

## Abstract

Feed-forward loops (FFLs) are thought to be one of the most common and important classes of transcriptional network motifs involved in various diseases. Enhancers are cis-regulatory elements that positively regulate protein-coding genes or microRNAs (miRNAs) by recruiting DNA-binding transcription factors (TFs). However, a comprehensive resource to identify, store, and analyze the FFLs of typical enhancer and super-enhancer FFLs is not currently available. Here, we present EnhFFL, an online database to provide a data resource for users to browse and search typical enhancer and super-enhancer FFLs. The current database covers 46 280/7000 TF-enhancer-miRNA FFLs, 9997/236 enhancer-miRNA-gene FFLs, 3 561 164/3 193 182 TF-enhancer-gene FFLs, and 1259/235 TF-enhancer feed-back loops (FBLs) across 91 tissues/cell lines of human and mouse, respectively. Users can browse loops by selecting species, types of tissue/cell line, and types of FFLs. EnhFFL supports searching elements including name/ID, genomic location, and the conservation of miRNA target genes. We also developed tools for users to screen customized FFLs using the threshold of *q* value as well as the confidence score of miRNA target genes. Disease and functional enrichment analysis showed that master miRNAs that are widely engaged in FFLs including TF-enhancer-miRNAs and enhancer-miRNA-genes are significantly involved in tumorigenesis. **Database URL:**http://lcbb.swjtu.edu.cn/EnhFFL/.

## Introduction

A disorder of network is the major cause of many diseases. The feed-forward loop (FFL), one of the most common network motifs, plays a key role in gene regulation and the development of diseases. A FFL consists of two input regulators and one output regulator which is jointly regulated by the two input regulators. FFLs consisting of transcription factors (TFs), microRNAs (miRNAs), and their joint target genes have been extensively studied. TFs, as master regulators of gene expression, perform an essential role in transcriptional regulation by binding to the transcription factor binding sites (TFBS). Previous studies found that c-Myc could activate the expression of miR-17-5p and miR-20a on human chromosome 13, and it was also confirmed that E2F1, a target of transcription factor c-myc, was negatively regulated by miR-17-5p and miR-20a. Therefore, a Myc-miR-17-5p/miR-20a-E2F1 FFL could be constituted to regulate c-Myc-mediated cellular proliferation.^1^ To date, many TF-miRNA-gene FFLs have been identified and confirmed to be associated with cancer, cardiovascular diseases, disorders of nervous system, etc.^2–6^

Enhancers act as cis-regulatory elements to positively regulate gene expression by recruiting DNA-binding TFs in a tissue-specific manner.^7^ They can activate the transcription of their target genes by recruiting TFs to perform functions on cell proliferation, development, and identification. Previous studies have reported that the enhancer target genes which encode lineage-specific TFs usually bind to enhancer regions to form various FFLs that function in the regulation of transcription and the maintenance of lineage.^8–10^ Recently, Suzuki *et al*.^11^ revealed that enhancers can regulate the expression of adjacent miRNAs and participate in the biological synthesis of Drosha/DGCR8-mediated primary miRNA, suggesting that the enhancer-miRNA regulatory relationships can participate in the entire transcription regulatory network as a new FFL form. The above studies^8–11, 12^ indicated that as a master regulator, enhancers could mediate diverse FFLs and play a crucial role in transcriptional regulation, development, and tumorigenesis, etc. However, to our knowledge, there is currently no comprehensive online resource to integrate enhancer-mediated regulatory relationships for the identification, analysis, storage, and visualization of enhancer FFLs. To date, some databases related to FFL have been built to study this information. For example, feed-forward loop-related databases like CircuitsDB,^13^ TransmiR,^14^ and FFLtool,^15^ and TF-target gene regulation databases such as hTFTarget^16^ and Cistrome.^17^ Nevertheless, CircuitsDB and Cistrome databases have stopped updating, and most of these focus only on the relationship between TF, gene, and miRNA, without involving the enhancer.

Here, we integrated the regulatory relationships among enhancers, TFs, miRNAs, and genes to construct EnhFFL, the first database for enhancer-related FFLs, which contains enhancer-tissues/cell lines of human and mouse. At present, EnhFFL has identified 46 280/7000 TF-enhancer-miRNA loops, 9997/236 enhancer-miRNA-gene loops, 3 561 164/3 193 182 TF-enhancer-gene loops, and 1259/235 TF-enhancer FBLs in human and mouse, respectively. EnhFFL provides a user-friendly way for users to query elements, including TFs, enhancers, miRNAs, and genes using name/ID or genomic location, etc., as well as to browse the loops in the selected tissue/cell lines. Additionally, it is also available to screen loops according to confidence score, conservation of miRNA target genes, or statistical significance. The regulatory pathways involved in the EnhFFL database provide plenty of reliable resources for further experiments in the study of transcriptional regulation, cell identification, development, and diseases. Bioinformaticians and computational biologists can also use our database for relevant regulatory network study and signal pathway analysis, as well as to integrate other regulatory elements they are interested in.

### Data source and database content

**Figure 1.**
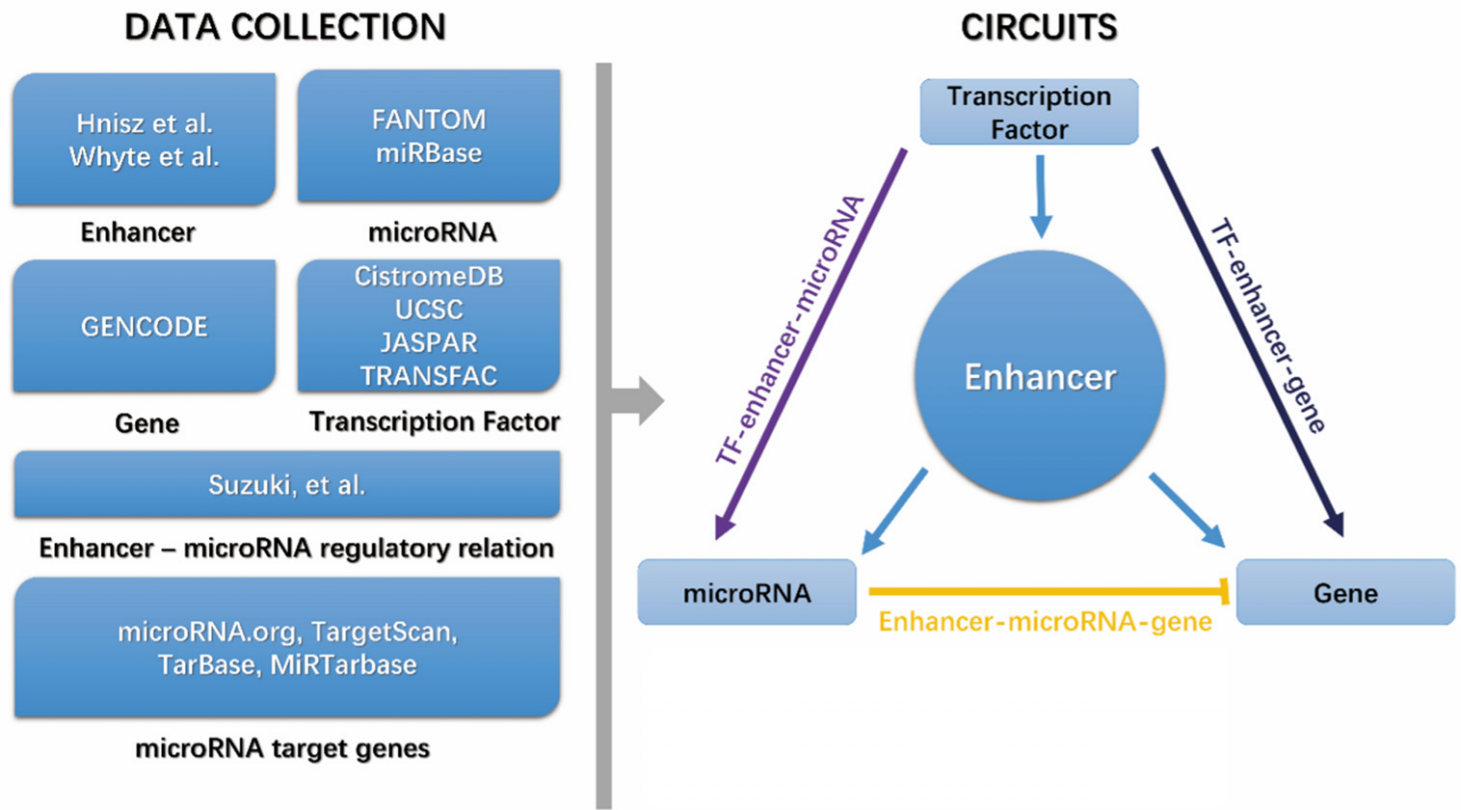
Main workflow of the database.

The enhancers were downloaded from the studies of Hnisz *et al*.^18^ and Whyte *et al*.,^8^ providing enhancers in 86 human tissue/cell lines (hg19) and five cell lines in mouse (mm9), respectively. Detailed information of data sources for the enhancer-miRNA regulation relationship, miRNAs, genes, and TFBS is listed in Supplementary Table S1. The database contains 16 cancer cell lines and 75 normal tissues/cell lines of human and mouse. In this study, a total number of 376 803 enhancers (332 723 typical enhancers and 44 080 super enhancers), 358 miRNAs, 164 TFs, as well as 14 774 genes are included for human. And, for mouse, there are 44 676 enhancers (42 563 typical enhancers and 2113 super enhancer), 161 miRNAs, 255 TFs, and 19 183 genes. As for the four types of loops, EnhFFL contains 46 280/7000 TF-enhancer-miRNA FFLs, 9997/236 enhancer-miRNA-gene FFLs, 3 561 164/3 193 182 TF-enhancer-gene FFLs, and 1259/235 TF-enhancer FBLs identified for human and mouse, respectively. The main workflow to get the detailed information is presented in Fig. 1.

### Identification of miRNA target genes

Experimentally validated microRNA target genes were obtained from miRTarBase v7.0^19^ and TarBase v8.0^20^ databases. We also collected the results predicted by TargetScan v7.0^21^ and microRNA.org v2010^22^ using default parameters. To integrate the results mentioned above, we manually calculated a target score to evaluate the confidence level of miRNA target gene prediction. The formula is as follows:


(1) \begin{eqnarray*} {\rm{S\ }} = {\rm{\ }}0.5\,{\rm{\times}}\,{N_i} + {N_c} \end{eqnarray*}

Here, ${N_i}$ represents the number of databases with consistent results for prediction of miRNA target genes, and ${N_c}$ represents the number of databases with experimentally verified results in which the miRNA-gene interaction exists. The range of *S* values is from 0.5 to 3.0, with a higher S score indicating a more reliable miRNA-gene relationship. The results showed that the *S* scores of most target genes were < 1 (Supplementary Fig. S1). With the purpose of reducing the false positive rate, only the relationships with target score > 1 were adopted for the following research.

### Integration of transcription factor binding site data

To determine the binding sites for the transcription factors, we downloaded the TF ChIP-seq data of corresponding tissue/cell line samples from CistromeDB^23^ as well as ENCODE.^24^ If there was more than one TFBS for a TF in specific tissue/cell line, we took the intersection of TFBSs from all corresponding data as the final TFBS result. If the TF ChIP-seq data were not available in the corresponding tissue/cell line, we adopted the Txn Factor ChIP track data, a track which shows regions of transcription factor binding derived from ENCODE ChIP-seq data across multiple tissue/cell lines. A score > 500 was reserved to screen highly conserved TFBSs across tissue/cell lines. As there were no available ChIP-seq data of TF for myotube cell, Th cell, and pro-B cell in mouse, their TFBSs were predicted using tfscan^25^ and MOODs^26^ based on the metrics from TRANSFAC^27^ and JASPAR,^28^ respectively.

### Identification of regulation of enhancer-gene, TF-enhancer, TF-gene, and TF-miRNA

A previous study showed that the distance between enhancers and promoters of their target genes fell mainly in the region of 100 kb.^29^ Therefore, TSS of genes within 100 kb upstream and downstream from the center of the enhancers were considered to be the target genes of enhancers. If the TFBS overlaps with the enhancer region, we regarded that this enhancer could be regulated by the TF. We also identified the enhancer-TF relationships by screening the TF-coding genes present in the enhancer-gene loops.

We took the regions 10 kb upstream and 1 kb downstream of miRNA TSS as the regulatory regions of miRNA promoter.^30^ Similarly, the regions 5 kb upstream and 1 kb downstream of gene TSS were identified as the regulatory regions of gene promoter.^31^ We considered that there was a TF-miRNA or TF-gene regulatory relationship if the regulatory region of miRNA/gene promoter overlapped with the TFBS. In addition, we collected different types of data to predict the relationship between enhancers, transcription factors, miRNAs, and target genes, and the classification of these data has been integrated into Supplementary Table S1.

### Identification of FFLs

The FFLs of TF-enhancer-miRNA, enhancer-miRNA-gene, and TF-enhancer-gene were built based on the regulatory relationships identified above. Taking TF-enhancer-miRNA as an example, for this type of FFL, the TF regulates the enhancer, and meanwhile, the TF and the enhancer also regulate at least one miRNA jointly. To construct FFLs, firstly, regulatory relationships among enhancers, miRNAs, and TFs previously identified were all integrated. Secondly, hypergeometric tests were performed on the candidate loops to ensure that they had statistical significance. The calculation formula is shown as below:


(2) \begin{eqnarray*} {\rm{\mathit{ P}\ }} = {\rm{\ }}1 - \mathop \sum \limits_{i\ = \ 0}^x \frac{{\left( {\begin{array}{@{}*{1}{c}@{}} k\\ i \end{array}} \right)\left( {\begin{array}{@{}*{1}{c}@{}} {M - k}\\ {N - i} \end{array}} \right)}}{{\left( {\begin{array}{@{}*{1}{c}@{}} M\\ N \end{array}} \right)}} \end{eqnarray*}

In the formula, *M* represents the total number of target miRNAs which are regulated by enhancers and TFs in the corresponding tissue/cell line. *N* and *k* are, respectively, the numbers of targets for a specific enhancer and a particular TF. *x* refers to the number of jointly regulated targets of the enhancer and TF mentioned above. Based on the *P* value of each TF-enhancer pair, *q* values with adjusted *P* values were obtained using the Benjamini-Hochberg procedure.^32^ Only pairs with *q* value less than the threshold of 0.05 were considered to be non-random pairs.

### Web interface

We developed a user-friendly web interface to help users to browse, search, and download the loops we identified. The web interface was split into five components: home, browse, search, download, and help. A brief introduction, workflow, and charts for statistics are presented on the home page. The browse page is composed of five drop-down menus, from which users can select the species, type of tissue/cell lines, specific tissue/cell line, type of loops, and conservation of miRNA target genes in which they are interested. As an aid, there are also sliders to set the threshold of *q* value as well as the confidence score of miRNA target genes. In the search page, each of the species is composed of four entries, including enhancers, miRNAs, genes, and transcription factors. Detailed choices for the conservation of miRNA target genes are also accessible. In addition, just as in the browse page, the threshold of *q* value and the confidence score of miRNA targets are also available.

### Browsing and searching the database

**Figure 2.**
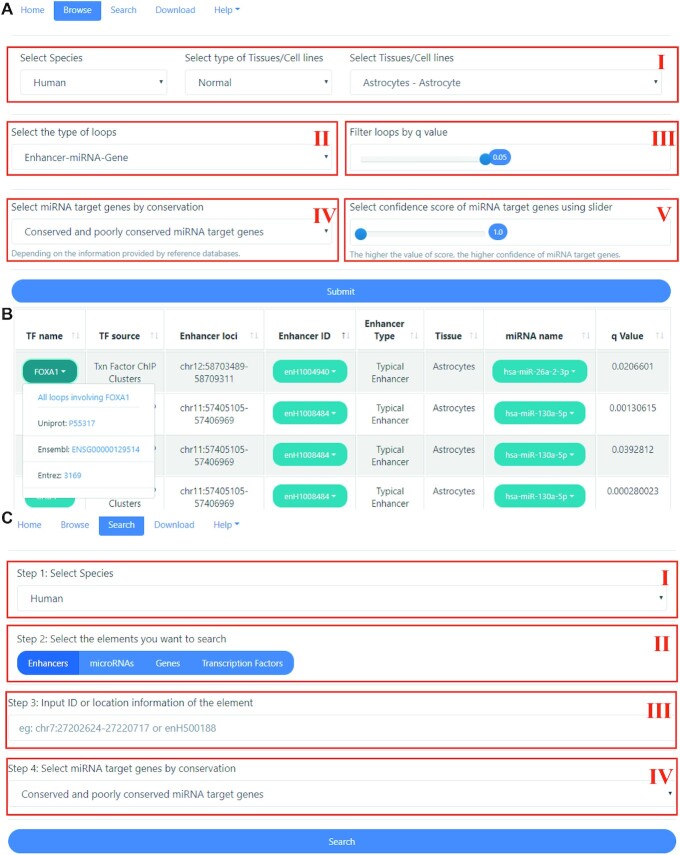
Usage example for the web interface. (A) Usage example for the browse page. The interface of browse page (Ⅰ–Ⅴ). In the browse page, there are options for species, types of tissue/cell lines, tissue/cell lines (Ⅰ), types of loops (Ⅱ), thresholds of q value (Ⅲ), as well as conservation (Ⅳ), and confidence scores of miRNA target genes (Ⅴ) available for users to screen loops in which they are interested. Confidence score range from 1.0 to 3.0, the higher the value of score, the higher confidence of miRNA target genes. (B) An example of a TF-enhancer-miRNA FFL result table (FOXA1-enH1004940-miR-26a-2-3p). By clicking on the drop-down lists of the three elements, users have access to more detailed information and external links. The page will redirect to the detailed page which shows all the loops that the element is involved in. Take FOXA1 as an example, by clicking the link ‘All loops involving FOXA1’, the page will turn to a new page which includes all types of loops that FOXA1 is involved in. (C) Usage example for the search page. Users can access results for elements by selecting the species (Ⅰ), choosing types of elements (Ⅱ), inputting Name/ID or genomic loci (Ⅲ), and customizing results for the conservation of miRNA targets( Ⅳ).

In the browse page, users can view the specific loops freely in only two steps. Firstly, users are supposed to select the tissue or cell line with the drop-down menu. Secondly, they can select the type of loops, including enhancer-miRNA-gene FFL, TF-enhancer-gene FFL, TF-enhancer-miRNA FFL, and TF-enhancer FBL. As an alternative, the threshold of *q* value can be set by slider to screen different levels of statistically significant FFLs. Considering that there are false-positive results in the prediction of miRNA target genes, EnhFFL provides choices of the conservation as well as the confidence score of miRNA target genes to help screen the enhancer-miRNA-gene loops of high quality (Fig. 2A).

Take TF-enhancer-miRNA FFLs in human astrocyte cells as an example, if users set the maximum *q* value to 0.05 and the minimum miRNA target confidence score to 1.0, and choose to show all the conserved and poorly conserved miRNA targets, the results will be presented as in Fig. 2B. Each row provides the information on elements that make up the TF-enhancer-miRNA FFL, and users can refine the results with a second query in the result table. For each enhancer, the type of enhancer (typical enhancer or super enhancer), genomic loci, and external link to UCSC browser are shown in the drop-down menu of the enhancer column. For miRNA, apart from the location information, TSS, external link to miRbase v20/v18^33^ was also available in the list. For TFs and genes, we provide links to Ensembl,^34^ Entrez Gene,^35^ Uniprot,^36^ and Entrez Gene.^35^ In addition, by clicking on the first line in the drop-down list, users can access the detailed page containing all types of loops in which the corresponding elements are involved (Fig. 2B).

There are four steps to search FFLs (Fig. 2C). Step 1, select the species. Step 2, select the type of elements including enhancers, miRNAs, genes, and transcription factors. Step 3, input the name/ID or genomic location of the element in which the users are interested. Step 4, select miRNA target genes by conservation when searching enhancer-miRNA-gene FFLs and TF-enhancer-miRNA FFLs.

### The master miRNAs of cancer FFLs significantly involved in tumorigenesis

The enhancer-miRNA regulation is a newly discovered regulatory network for miRNAs, but the function of the corresponding FFLs remains unclear. Therefore, we firstly investigated the master miRNAs that are widely engaged in FFLs including TF-enhancer-miRNAs and enhancer-miRNA-genes. Statistical analysis was performed to investigate the frequency of miRNAs involved in 16 cancer samples (Supplementary Fig. S2). We found that there were 17 master miRNAs, including hsa-miR-141-3p, hsa-miR-141-5p, hsa-miR-21-3p, hsa-miR-21-5p, hsa-miR-26a-2-3p, hsa-miR-26b-3p, hsa-miR-26b-5p, hsa-miR-29a-3p, hsa-miR-29a-5p, hsa-miR-30d-3p, hsa-miR-30d-5p, hsa-miR-339-3p, hsa-miR-339-5p, hsa-miR-33b-5p, hsa-miR-545-5p, hsa-miR-629-3p, and hsa-miR-629-5p, widely engaged in most of the samples (at least 14 out of 16), in particular hsa-miR-33b and hsa-miR-141 are engaged in all 16 samples. To explore the potential function of these miRNAs, we searched the HMDD database,^37^ a database of experimentally confirmed miRNA functions. The results showed that the 17 miRNAs mentioned above were widely engaged in tumorigenesis (Supplementary Table S2). For example, it is reported that miR-33b can inhibit metastasis of breast cancer by regulating expression of HMGA2, SALL4, and Twist1.^38^ It can also inhibit cell growth, invasion, and epithelial-mesenchymal transition, by suppressing Wnt/beta-catenin/ZEB1 signaling in lung cancer.^39^

## Conclusions

The EnhFFL database integrates regulatory relationships among enhancers, miRNAs, TFs, and genes to construct FFLs, a base branch of network motifs. Currently, the database covers 46 280/7000 TF-enhancer-miRNA FFLs, 9997/236 enhancer-miRNA-gene FFLs, 3 561 164/3 193 182 TF-enhancer-gene FFLs, and 1259/235 TF-enhancer feed-back loops (FBLs) across 91 tissue/cell lines of human and mouse, respectively. The database can provide lots of useful resources for biologists who work on regulatory research into enhancers, miRNA, and transcription factors. For the next releases, we will focus on collecting data from various species to construct a multi-species enhancer FFL database. In addition, the experimentally verified FFLs and TFBS from the literature will be added. We will continuously collect the latest data sets to keep the database up-to-date.

## Supplementary Material

pbab006_Supplemental_File
