# Supplementary material for: EnhFFL: A database of enhancer mediated feed-forward loops for human and mouse
Source: Precis Clin Med. 2021 Apr 14;4(2):129–35. doi: 10.1093/pcmedi/pbab006 (PMC8982537; doi:10.1093/pcmedi/pbab006)
Supplement: pbab006_Supplemental_File [file pbab006_supplemental_file.zip › Supplementary Table 1.docx]

Table 1. Sources of various types of data.

| Data Type | Source | Species | Version |
| --- | --- | --- | --- |
| TFBS ChIP-seq | CistromeDB(14) | Human/Mouse |  |
|  | ENCODE(15) | Human/Mouse |  |
|  | UCSC Txn factor track | Human |  |
| TFBS Prediction | JASPAR(16) | Mouse |  |
|  | TRANSFAC(17) | Mouse |  |
| Gene Annotation | GENCODE(18) | Human/Mouse | V19/Mm9 |
| Enhancer-miRNA regulation | Suzuki *et al*. (12) | Human/Mouse | hg19/Mm9 |
| miRNA Annotation | miRBase(19) | Human/Mouse | V20/ V18 |
| miRNA TSS | FANTOM5(20) | Human/Mouse |  |
| miRNA Target Gene | TargetScan(21) | Human/Mouse | Release 7.0 |
|  | miRTarbase(22) | Human/Mouse | Release 7.0 |
|  | TarBase(23) | Human/Mouse | V8.0 |
|  | microRNA.org(24) | Human/Mouse | 2010 Release |
