# Supplementary figures and images for: EnhFFL: A database of enhancer mediated feed-forward loops for human and mouse
Source: Precis Clin Med. 2021 Apr 14;4(2):129–35. doi: 10.1093/pcmedi/pbab006 (PMC8982537; doi:10.1093/pcmedi/pbab006)

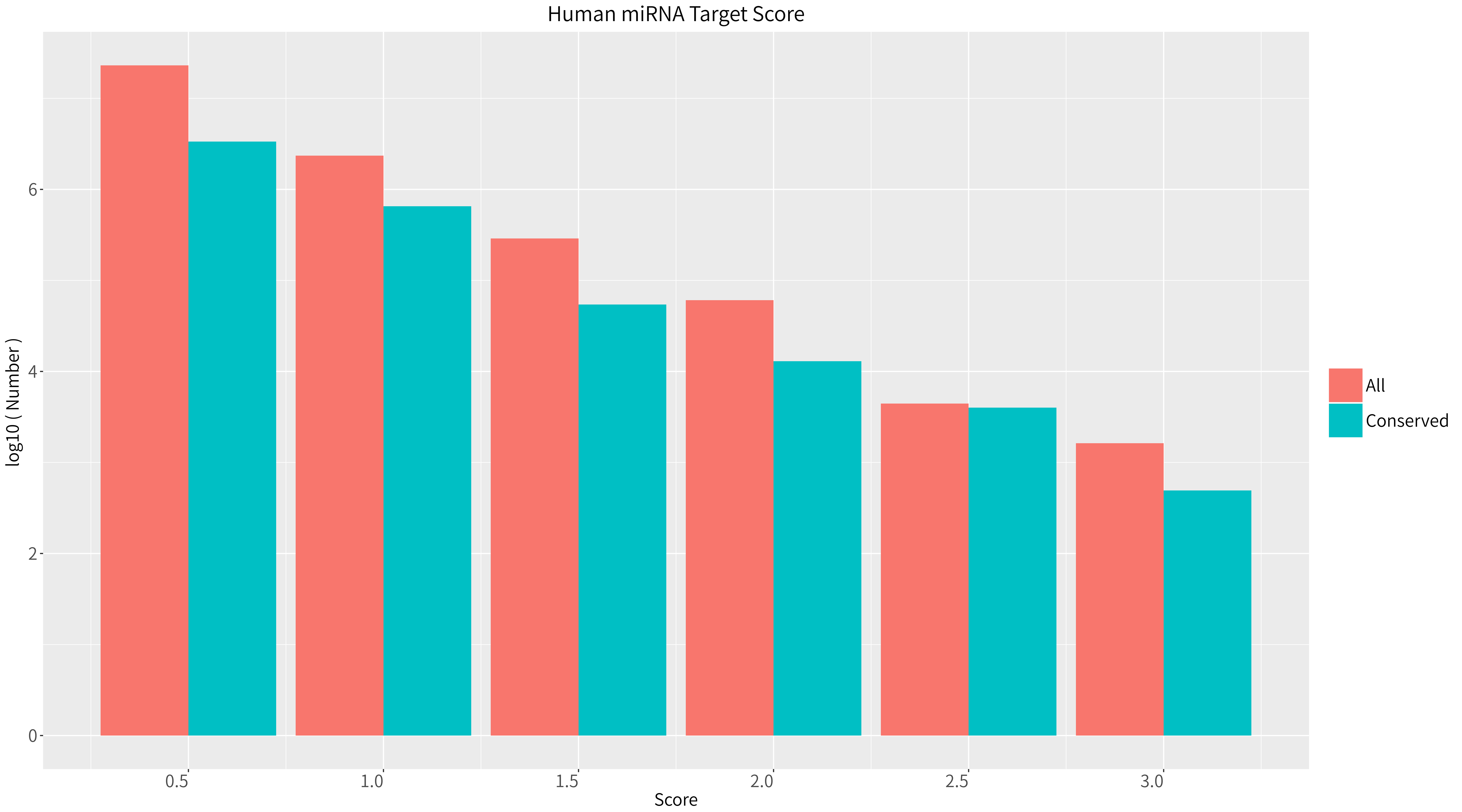

Supplement: pbab006_Supplemental_File [file pbab006_supplemental_file.zip › Supplementary Figure 1.tiff]

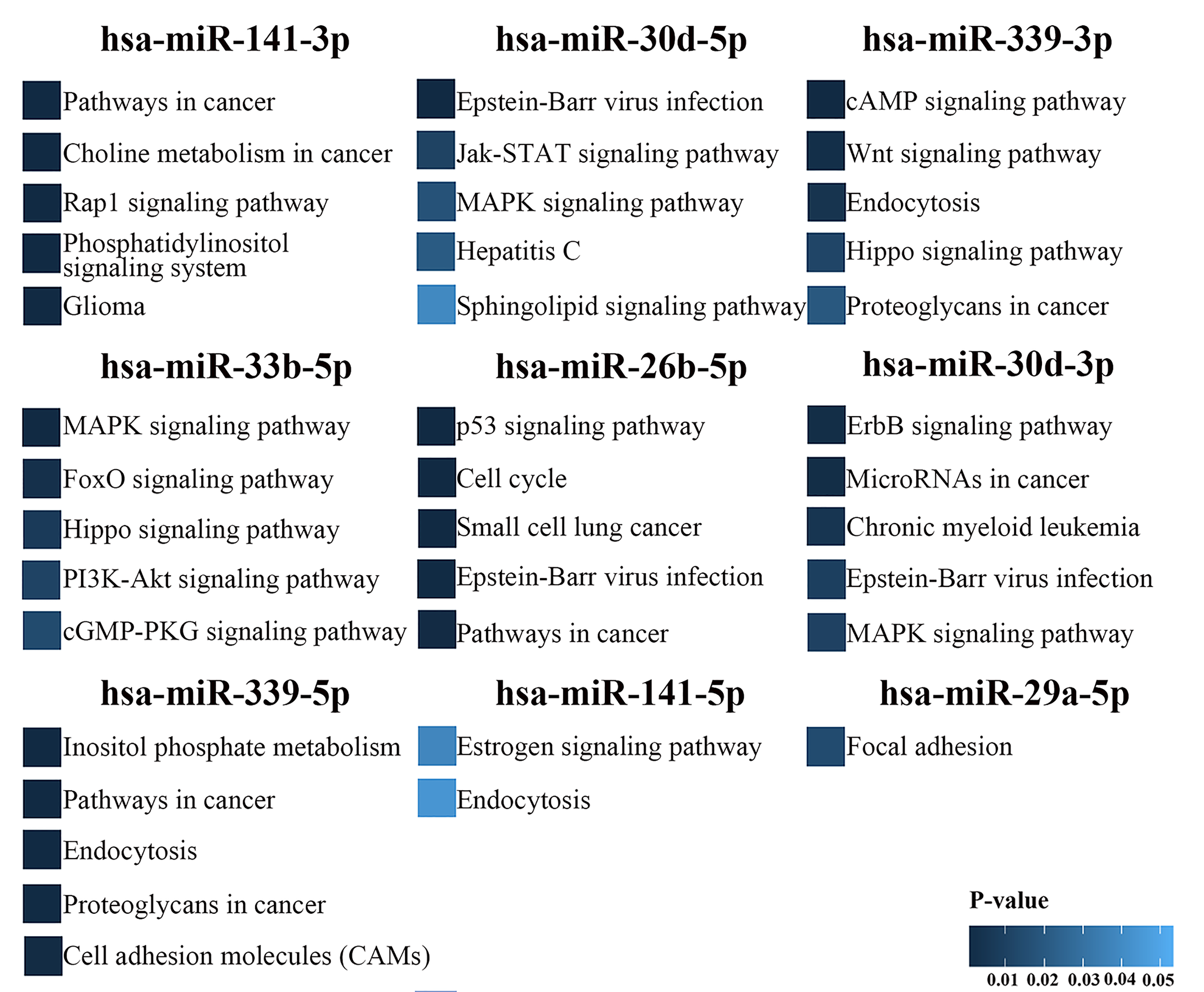

Supplement: pbab006_Supplemental_File [file pbab006_supplemental_file.zip › Supplementary Figure 2.tif]
